# Patterns of road traffic injury and potential consequences among patients visiting Hawassa University Comprehensive Specialized Hospital, Hawassa, Ethiopia

**DOI:** 10.1186/s13104-019-4192-5

**Published:** 2019-03-29

**Authors:** Bereket Duko, Fikru Tadesse, Zewdie Oltaye

**Affiliations:** Faculty of Health Sciences, College of Medicine and Health Sciences, Hawassa University, P.O. Box 1560, Awasa, Ethiopia

**Keywords:** Prevalence, Road traffic accident, Emergency department, Ethiopia

## Abstract

**Objective:**

Road traffic injury (RTI) is the leading cause of death among aged 15–29 years, although low and middle income countries only have half of the vehicles, they have 80% of road traffic related death. This study aimed to assess the probability of road traffic injury occurrence and potential consequences among patient visiting at emergency department of Hawassa University Comprehensive Specialized Hospital, Hawassa, Ethiopia. Retrospective cross-sectional study was conducted from March 8 to April 6/2018 among 350 patients who were recruited using systematic random sampling techniques. Binary logistic regression analysis was used for data analysis.

**Results:**

A total of 350 patients medical charts were reviewed at emergency department. The prevalence of road traffic accident was 40.9%. Being male (AOR = 1.84: 95% CI 1.11–3.09), being in age group of 20–29 (AOR = 2.58: 95% CI 1.14–5.84) and being in urban area of residence (AOR = 2.46; 95% CI 1.51–4.02) were significantly associated with road traffic accident. Conducting further research on road traffic injury and risk factors recommended.

## Introduction

Road traffic injury (RTI) is fatal or nonfatal accident that incurred as a consequence of a collision on a public road and pedestrians. As global focus of prevention and early intervention techniques have substantially reduced the burden of infectious diseases, unintentional injuries are increasing in significance as a public health problem. It is estimated that 1.2 million people are killed in road crashes every year. In addition to this, 50 million are injured worldwide [1].

In both developed and developing world, it becomes known causes of morbidity and mortality. Based on 2008 global burden of disease report, every year 5.1 million people died due to violence and injury [2]. Road traffic injuries are ranked 9th global cause of disability-adjusted life year lost and developing countries account over 80% of death globally due to road traffic accidents [3].

It has been the leading causes of trauma admission in the emergency unit of most hospitals. In developing countries, morbidity and mortality as a result of road traffic injury alarmingly rising. This is due to poor road and traffic infrastructure as well as the behaviors of road users. Studies showed that road traffic accident affects mainly male and those in age group of 15–44 years [4].

Although low and middle income countries have only 48% of worlds the registered vehicles, over 90% of road traffic injury occurs in these countries. Burden of road traffic injuries costs most countries 1–3% of their gross national product; without action, road traffic crashes are predicted to result in the death of around 1.9 million people annually by 2020 [5].

Different studies conducted in different countries on prevalence of road traffic accident injuries related death showed that 58% in Tunisia, 64% in Egypt, and 51% in Morocco. Being in age group of 15–59 and being male were factors which are associated with the injury [6].

The costs of fatality and injuries due to RTIs have a tremendous impact on social well-being and socio-economic development endeavors. Although it has high rate of occurrence resulting in high mortality and morbidity in Ethiopia, there are less studies done on similar issue in different set up. Therefore, this study aimed to assess the probability of road traffic injury occurrence and potential consequences among patient visiting at emergency department of Hawassa University comprehensive specialized Hospital, Hawassa, Ethiopia.

## Main text

### Study setting and population

Retrospective cross sectional study design was conducted in Hawassa University Comprehensive Specialized Hospital which is found in Hawassa City, Ethiopia. The hospital provides out and in patient service for about 18 million populations from South region and other neighboring regions. The emergency department of the hospital delivers services for 102,033 patients per year. In addition to this, this unit delivers service to 1800 injured patients per year. Single population proportion formula was used to calculate sample size using prevalence (p) = 50% with confidence interval of 95% and marginal error of 5%. A systematic random sampling technique was used to recruit study participant’s medical record. All eligible RTA injured patient’s medical records (charts) of patients who had visited the emergency department since March 2018 was included in the sample while incomplete medical records and died patients medical records were not included the study.

### Data collection instrument

Data was collected by four researchers who were given intensive primary and secondary data collection techniques. We used WHO injury surveillance checklist which was adapted for local setting that consists of items regarding the socio-demographic characteristics, severity, mechanism and consequences of accident cases. We had evaluated 6 month medical records was used from record of all patients visited emergency department as of March 2018. The card number was collected from in patient’s medical record (admitting, discharge log books and patient chart) from emergency room to get the main files from Central registration room. Using the medical record number (MRN) of the patient, a patient medical card which had all variables of interest was selected.

### Data processing and analysis

Data was checked for completeness and consistency. We had checked the collected data for completeness, consistency and accurate filling by the data collectors to assure the data quality. Then coded, cleaned and entered to EPI info version 7 and was analyzed by using SPSS version 22. Descriptive statistics was made to describe the socio-demographic characteristics of study participants. P-value less than 0.05 were considered as statistically significant. Binary logistic regression analysis was performed to identify associated factors. The strength of the association was presented by odds ratio and 95% confidence interval.

### Result

#### Socio-demographic characteristics of the respondents

A total of 350 patients medical record (charts) were included in the study. Among these, 206 (59.9%) were male, 70% were in age ranges of 20–49 years and 206 (58.9%) were living in urban area of residence (Table 1).

**Table 1:** Socio demographic characteristics of patients and Pattern of injury at Hawassa University Comprehensive Specialized Hospital, Hawassa, Ethiopia, 2018

Characteristics	Frequency (n)	Percent (%)
Age category		
< 20 years	42	12
20–29 years	118	33.7
30–39 years	78	22.5
40–49 years	48	13.7
50–59 years	26	7.4
> 60 years	38	10.9
Sex		
Male	206	58.9
Female	144	41.1
Ethnicity		
Sidama	122	34.9
Wolayta	29	8.3
Oromo	144	41.1
Gurage	25	7.1
Others	30	8.6
Residence		
Urban	206	58.9
Rural	144	41.1
Pattern of injury by type of vehicle (143)		
Motor bicycle crash	70	49
Public transport	24	16.8
Tricycle Bajaj	20	14
Mini-bus	16	11.2

#### Prevalence of road traffic accident among emergency department

**Fig. 1:**
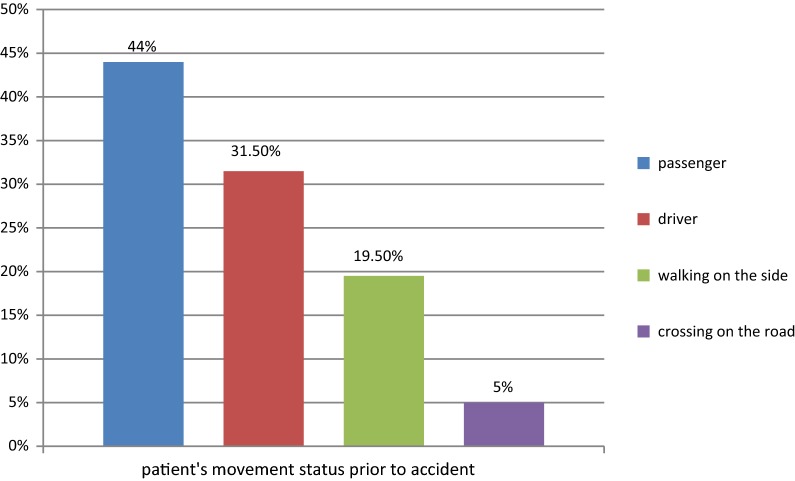
Patient’s movement status prior to the accident among patients visiting emergency department of Hawassa university comprehensive specialized hospital, 2018

The prevalence of road traffic accident in the current study was 40.9%. From those victims of road traffic accident, 106 (74.1%) were occurred by public transport they used, 27 (18.8%) pedestrian and 10 (6.9%) accident was occurred around street (Fig. 1). Regarding the reason of RTA, 66 (48.9%) was due to high speed, 46 (32.2%) was due to collision with other vehicles, 14 (9.8%) were failure to pass pedestrian and 17 (11.8%) were due to unknown reason which was not recorded in the patient chart. Regarding time of the accident, 48 (33.6%) was occurred in the morning, 51 (35.6%) in the evening and 16 (11.2%) was occurred overnight. The anatomical site of the injury was head which accounts 55 (38.5%), lower extremity 40 (27.9%), upper extremity 27(18.8%), chest injury 10 (6.7%) and others 11 (7.7%).

#### Factors associated with road traffic accident

Binary logistic regression analysis showed that being in age group of 20–29 years, being male and being in urban area of residence were significantly associated with road traffic accident victims (Table 2).

**Table 2:** Associated factor of RTA in emergency department of the hospital, Hawassa, Ethiopia, 2018

Variables	Road traffic accident		AOR, 95% CI	P-value
	Yes	No		
Age category				
< 20 years	18	24	2.57 (0.61–4.04)	0.35
20–29 years	64	54	*2.58 (1.14–5.84)*	*0.01*
30–39 years	22	56	0.75 (0.31–1.81)	0.52
40–49 years	23	25	2.08 (0.84–5.34)	0.12
50–59 years	22	4	0.44 (0.12–1.63)	0.22
≥ 60 years	8	30	*1*	
Sex				
Male	108	98	*1.84 (1.11– 3.09)*	*0.01*
Female	35	109	*1*	
Residence				
Urban	104	102	*2.47 (1.51–4.02)*	* < 0.01*
Rural	35	109	*1*	

1—reference category

Statistically significant variables are highlighted in italic

### Discussion

This study showed that the prevalence of road traffic injury was 40.9% (CI 38.86–42.95). The current study finding was higher than other studies in Addis Ababa [7, 8], in Jimma [9], in India [10], in Kenya [11]. On the other hand, the finding was lower than studies in Arbaminch [12] and in Tanzania [13]. The possible difference in the prevalence was might be due to difference in study design other studies used, sample size, study setting capacity, sample size and data collection instruments they used.

Being male was 1.84 more likely to have road traffic injury when compared to female. This finding is in agreement with other studies [12, 14, 15]. This might be due to males mainly involve in driving car, in comparison with females, males commonly found in urban streets, males work and they engage in high risk activities.

Victims were in age group of 20–29 years were more likely to have road traffic injuries. This is in line with other studies [9, 12, 16–19]. This might be explained the fact that this age group is the productive working years of life which they practice their life independently. In addition to this, in developing countries the large sizes of population are in this group of age.

Those study participants who were living in urban area were more likely to have road traffic injury. This is might be due to high traffic congestion in urban area, poor quality of the road and poor attitude in using cross roads.

### Conclusion

The prevalence of road traffic injury was high. Being male, being in age group of 20–29 years and living in urban place were found to be significantly associated with road traffic injury.

## Limitation of the study

The study used secondary data (medical records) of patient information. This limited not to asses all the contributing factors of road traffic injuries.
